# Culture-based study on the development of antibiotic resistance in a biological wastewater system treating stepwise increasing doses of streptomycin

**DOI:** 10.1186/s13568-018-0539-x

**Published:** 2018-01-25

**Authors:** Ganesh-Kumar Selvaraj, Zhe Tian, Hong Zhang, Mohanapriya Jayaraman, Min Yang, Yu Zhang

**Affiliations:** 1 State Key Laboratory of Environmental Aquatic Chemistry, Research Center for Eco-Environmental Sciences, Chinese Academy of Sciences, Beijing, 100085 China; 2 University of Chinese Academy of Sciences, Beijing, 100049 China

**Keywords:** Streptomycin (STM), Multi drug resistance (MDR), Antibiotic resistance genes (ARGs), Stepwise increasing STM doses

## Abstract

The effects of streptomycin (STM) on the development of antibiotic resistance in an aerobic-biofilm reactor was explored by stepwise increases in STM doses (0–50 mg L^−1^), over a period of 618 days. Totally 191 bacterial isolates affiliated with 90 different species were harvested from the reactor exposed to six STM exposures. *Gammaproteobacteria* (20–31.8%), *Bacilli* (20–35.7%), *Betaproteobacteria* (4.5–21%) and *Actinobacteria* (0–18.2%) were dominant, and their diversity was not affected over the whole period. Thirteen dominant isolates from each STM exposures (78 isolates) were applied to determine their resistance prevalence against eight classes of antibiotics. Increased STM resistance (53.8–69.2%) and multi-drug resistance (MDR) (46.2–61.5%) were observed in the STM exposures (0.1–50 mg L^−1^), compared to exposure without STM (15.3 and 0%, respectively). Based on their variable minimum inhibitory concentration results, 40 differentiated isolates from various STM exposures were selected to check the prevalence of nine aminoglycoside resistance genes (*aac*(*3*)-*II*, *aacA4*, *aadA*, *aadB*, *aadE*, *aphA1*, *aphA2*, *strA* and *strB*) and two class I integron genes (*3*′-*CS* and *IntI*). STM resistance genes (*aadA*, *strA* and *strB*), a non-STM resistance gene (*aacA4*) and integron genes (*3*′-*CS* and *Int1*) were distributed widely in all STM exposures, compared to the exposure without STM. This new culture-based stepwise increasing antibiotic approach reveals that biological systems treating wastewater with lower STM dose (0.1 mg L^−1^) could lead to notably increased levels of STM resistance, MDR, and resistant gene determinants, which were sustainable even under higher STM doses (> 25 mg L^−1^).

**Electronic supplementary material:**

The online version of this article (10.1186/s13568-018-0539-x) contains supplementary material, which is available to authorized users.

## Introduction

The broad use of antibiotics in the fields of medical therapy, animal husbandry, and plant disease control during the past 65 years has resulted in the rapid growth and global prevalence of antibiotic resistance. In recent years, municipal wastewater treatment plants (WWTPs) have been considered as the main source for the development of antibiotic resistant bacteria (ARB) and antibiotic resistance genes (ARGs), as they consist of a mixed bacterial community maintained by rich nutrient environments, and experience repeated contamination by resistant bacteria and exposure to various antibiotics (Gallert et al. 2005; Novo and Manaia 2010; Rizzo et al. 2013; Michael et al. 2013; Guo et al. 2017; Mao et al. 2015; Rodriguez-Mozaz et al. 2015; Aubertheau et al. 2017). In comparison with municipal WWTPs, however, antibiotic production WWTPs could become a hotspot for the development of antibiotic resistance due to the presence of much higher concentrations of antibiotics (Pruden et al. 2013; Ashbolt et al. 2013). It is important, therefore, to know the threshold concentration of antibiotics that leads to antibiotic resistance.

Our previous study showed that the presence of high concentrations of antibiotics (oxytetracycline, > 19.5 mg L^−1^) in a biological WWTP could lead to the occurrence of tetracycline resistance and MDR in treated wastewater and downstream rivers (Li et al. 2010). Similar results have also been observed in antibiotic production WWTPs accepting penicillin and spiramycin production wastewater (Li et al. 2009, 2011; Liu et al. 2014). However, most of these studies have been derived from investigative results of full-scale real WWTPs, and do not reveal the influence of antibiotic concentrations. Recently some effort has been made to investigate the influence of minimum antibiotic concentrations on the transfer of MDR under controlled experimental conditions. Under such studies, concentrations of tetracycline and sulfamethoxazole, as low as 10–100 µg L^−1^ were found to promote MDR through the horizontal dissemination of mobile resistance elements (Jutkina et al. 2016; Kim et al. 2014). However, pure cultures and overnight culture times have often been adopted for these experiments, which may not be able to reveal the impact of antibiotic concentrations on the wastewater treatment bacteria.

In the current new approach, a biofilm-type wastewater treatment reactor was constructed to treat synthetic wastewater under stepwise increasing doses of STM (0, 0.1, 1, 5, 25 and 50 mg L^−1^) over a period of 618 days, with a control system (no antibiotics) operated in parallel. Streptomycin is one of the first broad-spectrum antibiotics to be utilized for the control of infectious human and plant diseases. Although STM resistance mechanisms have been proposed in many clinical bacteria, plant and animal pathogens (De Leon Door et al. 2013; Sundin et al. 1995; Sundin 2002; Um et al. 2016), the resistance spectra in wastewater communities are still poorly understood. In additions, the culture-based wastewater bacterial responses against stepwise increasing doses of STM have not been studied. In this study, wastewater bacterial strains were isolated from six different STM exposures, and the impacts of antibiotic concentrations on wastewater bacterial diversity and the characteristics of antibiotic resistance were assessed using bacterial enrichment method, sequencing and, MIC assay. The presence and distribution of 11 antibiotic genetic determinants from individual wastewater bacterial isolates were determined by the conventional polymerase chain reaction (PCR). The results of this new culture-based stepwise increasing STM approach will enable us to better understand the risks accompanying antibiotic wastewater treatment.

## Materials and methods

### Sampling source

Two lab scale wastewater reactors (15 × 10 × 29.5 cm in size) were constructed with an effective volume of 2 L, and were each filled with 18 fiber balls as bio-carriers (Additional file 1: Fig. S1). Activated sludge from a municipal wastewater treatment plant in Beijing was used as the initial seeding inoculum. Glucose, tryptone, starch and sodium-carboxymethyl cellulose were used as the mixed carbon sources for the synthetic wastewater (Additional file 1: Table S1). Wastewater was fed into the reactors with a hydraulic retention time (HRT) of 24 h at room temperature (20 ± 5 °C). After the start-up period (1 month), one reactor was used to treat wastewater containing STM [streptomycin sulfate (95%), TCI, Shanghai, China] and the other was used as the control to treat wastewater without STM. The dosage of STM was increased stepwise in six exposures (0, 0.1, 1, 5, 25 and 50 mg L^−1^), with each exposures maintained for at least 14 weeks. The stability of wastewater treatment performance was confirmed by monitoring the effluent ammonium concentration. Increases in the STM dose were implemented only when the reactor had operated at an effluent ammonium concentration below 1 mg L^−1^ for over 1 month. The whole experimental period spanned 618 days including the start-up period. At the end of each STM exposure, two plastic balls were taken out of the reactor for biological analysis and the same number of fresh balls was put into the reactors. Each ball was shaken with 50 mL of phosphate buffer saline (PBS) and the solution was centrifuged at 10,000 rpm for 10 min at 4 °C. The same supernatant was used to wash the balls repeatedly until they returned to their original color. The processed sludge samples were stored at – 80 °C as a 30% glycerol stock solution for future use.

### Bacterial enrichment and purification

Ten-fold serial dilutions were performed for the processed sludge samples collected from all six exposures of the STM reactor and control reactor. The frozen sludge samples were firstly thawed gradually and homogenized by an ultra-Turrax disperser (IKA, T10 basic S25-unit). Triplicate non-selective media including TSA and R2A (Difco, France), were simultaneously prepared and inoculated by 0.1 ml aliquots of diluted sludge samples. Inoculated plates (As one, Shanghai, China) were incubated at 30 °C for 12–48 h. Bacteria with different morphologies were re-streaked at least three times on fresh corresponding media to obtain pure cultures. All cultivable bacteria were thoroughly screened and purified from all the STM exposures in biological reactor. Colony forming units (CFUs) of the various sludge samples were simultaneously determined on the TSA medium and microbial purity was checked by the Gram’s stain method. Purified bacteria from all STM exposures were stored as 30% glycerol stock solutions in the corresponding media at – 80 °C for future analysis.

### 16S rRNA gene analysis

To identify the bacterial isolates from all six STM exposures, genomic DNA was extracted from all purified bacteria and the 16S rRNA genes were amplified using universal primers (27F and 1492R). Bacterial genomic DNA was harvested using a TIANamp bacteria DNA kit, and the DNA concentration was determined using a Nanodrop 1000 spectrophotometer (Nanodrop, USA). Genomic DNA (50 ng) was used as the template DNA for the PCR mixture. The PCR mixture (25 µL) contained 1× PCR buffer (MgCl_2_^+^), 2.5 mM dNTPs mix, 0.5 µM of each primer, and 1 U of Taq DNA polymerase (Takara, Bio Inc, Shiga, Japan). The 16S rRNA-PCR conditions were as follows: initial denaturation at 94 °C for 10 min, followed by 30 cycles of denaturation at 94 °C for 1 min, annealing at 52 °C for 1 min, extension at 72 °C for 2 min, and a final extension at 72 °C for 10 min. After the PCR, the amplified PCR products were resolved on 1% agarose (Biowest, Hong Kong) gel.

### Sequencing and phylogenetic analysis

The purified PCR products were sequenced using an ABI3730 automated sequencer (Invitrogen, Shanghai, China). Bacterial 16S rRNA gene sequences were analyzed manually using Bioedit software. Sequenced DNA was compared using the Ribosomal Database Project (RDP) and GenBank-National Center for Biotechnology Information (NCBI) database, and sequence similarities above 99% were considered identified for species the level and above 98% for the genus level. The MEGA6 (Tamura et al. 2013) and Clustal W (Larkin et al. 2007) software were used to draw dendrograms of 90 different isolated bacterial strains. Bacterial phylogeny was tested using neighbor-joining and statistical analysis was done by the bootstrap method. Bootstrap analysis was used to determine the confidence values of phylogenetic tree nodes using 1000 replicates (Felsenstein 1985). The partial 16S rRNA gene sequences of isolates were deposited in NCBI, GenBank under various Accession Numbers (KY087979, KY087986–87, KY087989–90, KY392999–15, KY393017–36, KY393045–54, and KY393058–95).

### Minimum inhibitory concentration (MIC) assay

A total of 40 different bacterial strains (78 bacterial isolates, 13 from each STM exposure) were selected for testing of antibiotic resistance prevalence based on their dominance in all six STM exposures at the genus level. Determinations of MICs described in this protocol were in accordance with the recommendations given by the Clinical and Laboratory Standards Institute (CLSI 2016). Among the nine antibiotics (including eight different classes of antibiotics), the Etest gradient method was applied for azithromycin (AZ), ceftazidime (TZ), enrofloxacin (EF), sulfamethoxazole (SX), tetracycline (TC), tobramycin (TM), tigecycline (TGC), and ertapenem (ETP) (Additional file 1: Table S4). The Etest strips were purchased from Biomerieux (France). Micro-dilution (96-well-plate method) was applied only for STM, since Etest strip for this antibiotic was not commercially available. Overnight Mueller Hinton (MH) broth (Oxoid, England) culture was prepared from a single colony and its turbidity was adjusted in accordance with the 0.5 McFarland standard solutions. This diluted broth culture was used as the inoculum for both tests. The bacterial lawn was prepared on the MH plates (duplicates) using a cotton swab and the corresponding Etest strip was applied on the inoculated MH agar surface. Finally, air-dried inoculated plates were incubated at 30 °C for 24 h. For the 96-well-plates, the same diluted broth culture (75 µL) was loaded in each well of the micro-titer plates (in triplicate) containing STM solutions (75 µL) and incubated at 30 °C for 24 h (Andrews 2001). Since no elaborated CLSI guidelines are available for the environmental non-pathogenic bacteria, the antibiotic break point limits for MIC interpretation are scarce. Thus, the MIC patterns were grouped as sensitive (0.016–12 μg mL^−1^), resistant (13–64 μg mL^−1^) and highly resistant (64 to > 256 μg mL^−1^) (Lundstrom et al. 2016; Popowska et al. 2012). However a cut-off value for STM (> 8 μg mL^−1^) among *E. coli* was considered for STM resistance in this study (Sunde and Norstrom 2005).

### Screening of antibiotic genetic determinants

The presences of nine aminoglycoside resistance genes (*aac*(*3*)-*II*, *aacA4*, *aadA*, *aadB*, *aadE*, *aphA1*, *aphA2*, *strA* and *strB*), one clinical class I integron gene (*3*′-*CS*) and one class I integrase gene (*IntI*) were screened from 11 different bacterial strains (including 40 isolates from the six STM exposures), which exhibited variable MIC values with the increase in STM doses. Details of the specific primers used in this study are listed in Additional file 1: Table S6. The bacterial DNA template (~ 50 ng) was used and the PCR conditions were applied as described above (“16S rRNA gene analysis” section). However, the annealing temperature varied based on the specific primers used. After the PCR reactions, specific gene products were analyzed on 1.2% agarose gel stained with ethidium bromide. The sequenced resistance genes were checked by BLASTN in, GenBank, NCBI.

## Results

### Culture-based bacterial diversity

**Fig. 1:**
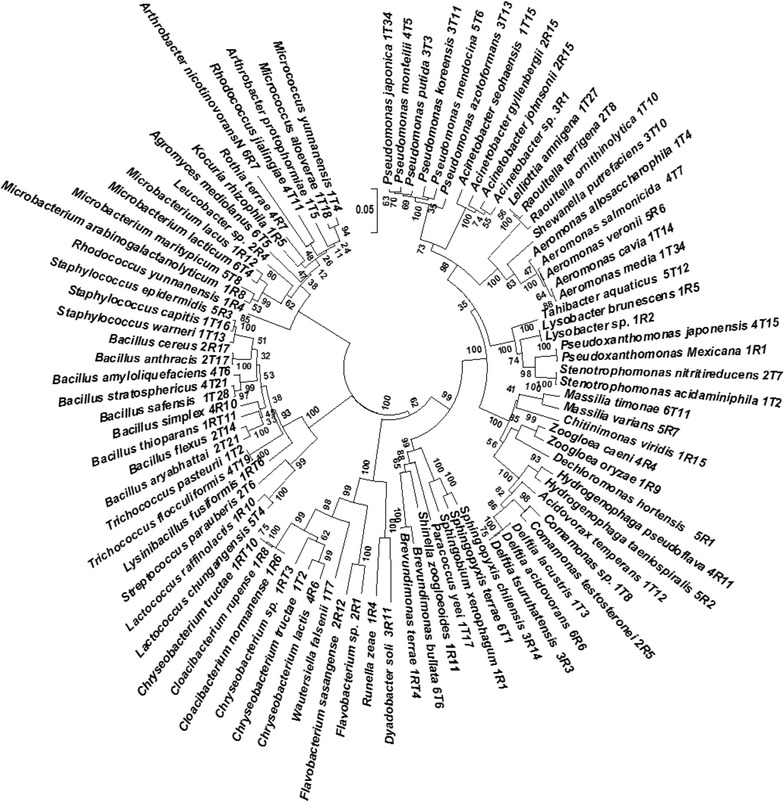
Neighbor-joining phylogenetic tree showing the clustering of the 16S rRNA gene sequences of bacterial strains isolated from various STM exposures in biological reactors. The constructed tree was tested by the bootstrap method and bootstrap values are depicted adjacent to each node. The 0.02 scale bar indicates the nucleotide substitution level

**Fig. 2:**
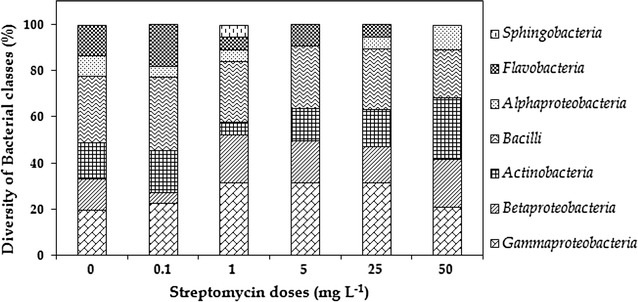
Distribution of bacterial classes harvested from the long-term wastewater reactor treated with stepwise-increasing doses of streptomycin

A total of 191 purified bacterial isolates including 56, 28, 26, 29, 26 and 26 isolates were harvested from the 0, 0.1, 1, 5, 25 and 50 mg L^−1^ STM exposures, respectively (Additional file 1: Table S2). A total of 90 different bacterial species including 7 classes, 44 genera and 25 families, were harvested from all six exposures of the STM reactor (Table 1), with the phylogenetic relationships of the total bacterial species illustrated in Fig. 1. *Gammaproteobacteria* (20–31.8%), *Bacilli* (20–35.7%), *Betaproteobacteria* (4.5–2%) and *Actinobacteria* (0–18.2%) were dominant in all six STM exposures (0–50 mg L^−1^) (Fig. 2). The remaining distributed bacterial classes were *Alphaproteobacteria* (0–13.3%), *Flavobacteria* (0–18.2%) and *Sphingobacteria* (0–5.2%). Overall, bacterial genera such as *Bacillus*, *Pseudomonas*, *Aeromonas*, *Microbacterium* and *Acinetobacter* were dominant, and bacterial species such as *Aeromonas veronii*, *Bacillus anthracis*, *Chryseobacterium lactis*, *Comamonas testosteroni*, *Lactococcus chungangnensis* and *Microbacterium maritypicum* were predominantly present in almost all STM exposures. Similar bacterial diversity was observed in the control reactor (Additional file 1: Table S3) without STM (0 mg L^−1^). Therefore those stains from the control system were not used for further analysis (data not included). Principal coordinates analysis (PCoA) showed that bacterial community in the STM exposures such as 5, 25 and 50 mg L^−1^ were clustered near by locations and its biodiversity was differed significantly with 0 and 0.1 mg L^−1^ STM exposures (Additional file 1: Fig. S2). From both the control and STM reactor, the approximate CFU in the TSA media ranged from (3.2 ± 1.8) × 10^4^ to (5.4 ± 1.4) × 10^4^ (average of triplicate plates).

**Table 1:** Phylogenetic affiliation of bacterial isolates harvested from stepwise increasing doses of STM treating aerobic-biofilm reactor

Bacterial classes	Bacterial families	Bacterial name	Distribution in STM stages (mg L^−1^)
*Actinobacteria*	*Microbacteriaceae*	*Agromyces mediolanus*	50
		*Leucobacter* sp.	0
		*Microbacterium arabino*	0, 1
		*Microbacterium lacticum*	0.1, 50
		*Microbacterium lacus*	0
		*Microbacterium maritypicum*	0.1, 5, 25, 50
	*Micrococcaceae*	*Arthrobacter nicotinovorans*	50
		*Kocuria rhizophila*	0, 0.1
		*Micrococcus aloeverae*	0, 1
		*Micrococcus yunnanensis*	0
		*Rothia terrae*	5
	*Nocardiaceae*	*Rhodococcus jialingiae*	5
		*Rhodococcus yunnanensis*	0
*Alphaproteobacteria*	*Caulobacteraceae*	*Brevundimonas bullata*	50
		*Brevundimonas terrae*	0
	*Rhizobiaceae*	*Shinella zoogloeoides*	0
	*Rhodobacteraceae*	*Paracoccus yeei*	0, 0.1
	*Sphingomonadaceae*	*Sphingobium xenophagum*	0
		*Sphingopyxis chilensis*	1, 25
		*Sphingopyxis terrae*	50
*Bacilli*	*Bacillaceae*	*Bacillus amyloliquefaciens*	0, 5
		*Bacillus anthracis*	0, 0.1, 1, 5, 25, 50
		*Bacillus aryabhattai*	0, 0.1
		*Bacillus cereus*	0.1, 1
		*Bacillus flexus*	0.1
		*Bacillus safensis*	0, 0.1
		*Bacillus simplex*	5
		*Bacillus stratosphericus*	5, 25
		*Bacillus thioparans*	0
		*Bacillus toyonensis*	0
		*Lysinibacillus fusiformis*	0
	*Carnobacteriaceae*	*Trichococcus flocculiformis*	0
		*Trichococcus pasteurii*	0
	*Staphylococcaceae*	*Staphylococcus capitis*	0
		*Staphylococcus epidermidis*	1
		*Staphylococcus warneri*	0
	*Streptococcaceae*	*Streptococcus parauberis*	0
*Betaproteobacteria*	*Burkholderiaceae*	*Chitinimonas viridis*	1, 50
	*Comamonadaceae*	*Acidovorax temperans*	0
		*Comamonas* sp.	1
		*Comamonas testosteroni*	0, 0.1, 1, 5
		*Delftia acidovorans*	5, 50
		*Delftia* sp.	0
		*Delftia tsuruhatensis*	1
		*Hydrogenophaga pseudoflava*	5
		*Hydrogenophaga taeniospiralis*	25
	*Oxalobacteraceae*	*Massilia timonae*	50
		*Massilia varians*	25, 50
	*Rhodocyclaceae*	*Dechloromonas* sp.	25
		*Zoogloea caeni*	5
	*Streptococcaceae*	*Lactococcus chungangensis*	0, 0.1, 1, 5, 25, 50
		*Lactococcus raffinolactis*	0, 1
*Flavobacteria*	*Cytophagaceae*	*Runella zeae*	0
	*Flavobacteriaceae*	*Chryseobacterium* sp.	0.1, 1
		*Chryseobacterium lactis*	0, 0.1, 1, 5, 25
		*Chryseobacterium rhizoplanae*	0
		*Cloacibacterium normanense*	0
		*Cloacibacterium rupense*	0
		*Flavobacterium hibernum*	0.1, 5
		*Flavobacterium sasangense*	0.1
		*Wautersiella falsenii*	0
*Gammaproteobacteria*	*Aeromonadaceae*	*Aeromonas caviae*	5
		*Aeromonas allosaccharophila*	0, 25, 50
		*Aeromonas media*	0
		*Aeromonas salmonicida*	5
		*Aeromonas veronii*	0, 0.1, 25
	*Enterobacteriaceae*	*Lelliottia amnigena*	0
		*Raoultella ornithinolytica*	0.1
		*Raoultella terrigena*	1, 25, 50
	*Moraxellaceae*	*Acinetobacter gyllenbergii*	0.1
		*Acinetobacter johnsonii*	0.1
		*Acinetobacter seohaensis*	0
		*Acinetobacter* sp.	1, 5, 50
	*Pseudomonadaceae*	*Pseudomonas azotoformans*	5
		*Pseudomonas japonica*	0
		*Pseudomonas koreensis*	1
		*Pseudomonas mendocina*	25
		*Pseudomonas monteilii*	5
		*Pseudomonas putida*	1
	*Shewanellaceae*	*Shewanella putrefaciens*	1
	*Xanthomonadaceae*	*Lysobacter brunescens*	0
		*Lysobacter* sp.	0
		*Pseudoxanthomonas japonensis*	5, 50
		*Pseudoxanthomonas mexicana*	0, 5, 25, 50
		*Pseudoxanthomonas* sp.	50
		*Stenotrophomonas* sp.	0.1
		*Stenotrophomonas acidaminiphila*	0, 0.1
		*Tahibacter aquaticus*	5, 25, 50
*Sphingobacteria*	*Cytophagaceae*	*Dyadobacter* sp.	1

### Determination of MICs

**Fig. 3:**
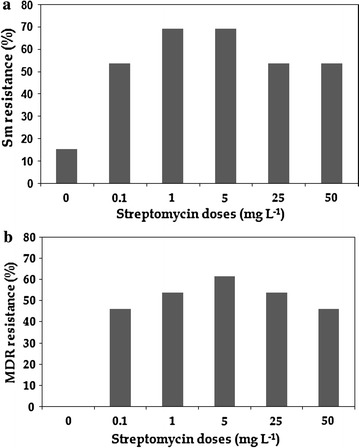
MIC assay showing the prevalence of bacterial streptomycin resistance (**a**) and MDR (**b**) in the various streptomycin exposures. A total of 78 dominant bacterial isolates (13 isolates from each exposure) and nine antibiotics were used for the MIC assay. The number of resistant strains among the total strains was taken for the calculation of resistance prevalence (%)

From the MIC results, 53.8–69.2% of STM resistance was observed in the presence of STM doses of 0.1–50 mg L^−1^ (Fig. 3a), whereas only 15.4% of STM resistance was observed in the exposure without STM (0 mg L^−1^). Bacterial strains such as *Aeromonas allosaccharophila*, *Aeromonas veronii*, *Chryseobacterium lactis*, *Comamonas testosteroni*, *Pseudoxanthomonas mexicana*, *Acinetobacter* sp., *Microbacterium maritypicum*, *Microbacterium lacticum*, *Raoultella terrigena* and *Sphingopyxis chilensis* exhibited highly resistance to streptomycin (> 1024 µg mL^−1^) in the corresponding STM exposures (Additional file 1: Table S5). In other hand, *Bacillus amyloliquefaciens*, *Bacillus anthracis*, *Bacillus cereus*, *Bacillus stratosphericus*, *Brevundimonas bullata*, *Trichococcus flocculiformis* and *Leucobacter* sp. showed least resistance to the STM (< 8 µg mL^−1^).

The capability for MDR was 46.2–61.53% among the isolates harvested from the 0.1 to 50 mg L^−1^ STM exposures, whereas there was no capability of MDR observed in the exposure without STM (Fig. 3b). Bacterial isolates including *Aeromonas allosaccharophila*, *Chryseobacterium lactis*, *Comamonas testosteroni* and *Microbacterium maritypicum* exhibited resistance to at least four different antibiotics, simultaneously. The bacterial isolates from various STM exposures exhibited co-resistance to azithromycin, ceftazidime, sulfamethoxazole, tobramycin and tetracycline with a ratio of 7.7–38.4%, whereas no or low co-resistance (0–7.7%) was observed for the antibiotics including enrofloxacin, tigecycline or ertapenem (Table 2; Additional file 1: Fig. S3).

**Table 2:** Activities of nine antibiotics against bacterial isolates harvested from stepwise increased STM exposures

Antibiotics	Antibiotic range (µg mL^−1^)	Prevalence of antibiotic resistance (%)^a^					
		0 mg L^−1^, STM	0.1 mg L^−1^, STM	1 mg L^−1^, STM	5 mg L^−1^, STM	25 mg L^−1^, STM	50 mg L^−1^, STM
AZ	0.016–256	0	38.4	38.4	30.8	23.0	23.0
TZ	0.016–256	7.7	23.0	38.4	23.0	15.4	15.4
EF	0.002–32	0	0	0	0	0	0
ETP	0.064–1024	0	0	7.7	7.7	7.7	7.7
SX	0.002–32	7.7	15.4	30.8	30.8	23.0	0
TC	0.016–256	0	23.0	30.8	30.8	30.8	15.4
TGC	0.016–256	0	0	0	0	0	0
TM	0.016–256	7.7	7.7	7.7	15.4	15.4	30.8
Sm	1–1024	15.4	53.8	69.2	69.2	53.8	53.8

*AZ* azithromycin, *TZ* ceftazidime, *EF* enrofloxacin, *ETP* ertapenem, *SX* sulfamethoxazole, *TC* tetracycline, *TGC* tigecycline, *TM* tobramycin, *STM* streptomycin

^a^A total of 40 different bacterial strains (78 bacterial isolates, i.e. 13 isolates from each Sm stage) were used for MIC assay and the percentage of antibacterial resistance was calculated by the number of resistant strains among the total stains

### Prevalence of antibiotic resistance genes and class I integron genes

Amino glycoside resistance genes *strA*, *strB*, *aadA* and *aacA4* (83.3–100%), and integron genes *IntI* and* 3*′-*CS* (97.5–100%) were widely observed among the strains harvested from the 0.1 to 50 mg L^−1^ STM exposures (Table 3; Additional file 1: Table S7), whereas STM resistance genes *strA* and *strB* (50.0%) and integron genes *IntI* (30%) and* 3*′-*CS* (70%) were also observed in the exposure without STM. In addition, other amino glycoside genes including *aac*(*3*)-*II*, *aphA1*, *aphA2*, *aadE* and *aadB*, were not notably distributed (0–17.5%) among the various STM exposures. In particular, the *aadE* gene (confers resistance to STM and spectinomycin) was observed only in *Lactococcus chungangensis* (1–50 mg L^−1^), whereas none of the STM resistant strains contained the *aac*(*3*)-*II* gene (confers resistance to gentamycin, tobramycin and kanamycin). In this study, *Lactococcus chungangensis* and *Stenotrophomonas acidaminiphila* exhibited the maximum number of amino glycoside resistance genes (six), whereas *Paracoccus yeei*, *Lactococcus raffinolactis*, *Pseudoxanthomonas mexicana* and *Chryseobacterium lactis* contained five amino glycoside resistance genes (Additional file 1: Fig. S4) and *Aeromonas veronii* and *Comamonas testosteroni* contained three amino glycoside resistance genes.

**Table 3:** Distributions of amino glycoside resistant genes and class I integron genes among the STM resistant isolates harvested from various STM exposures of aerobic-biofilm reactor

S. no	Bacterial strains name	0 mg L^−1^, STM	0.1 mg L^−1^, STM	1 mg L^−1^, STM	5 mg L^−1^, STM	25 mg L^−1^, STM	50 mg L^−1^, STM
1	*Aeromonas allosaccharophila*	*_*				*aacA4*, *strA*, *strB*, *Int1*, *3*′-*CS* (5)	*aacA4*, *aadA*, *strA*, *strB*, *Int1*,* 3*′-*CS* (6)
2	*Aeromonas veronii*	*_*	*aacA4*, *aadA*, *Int1*,* 3*′-*CS* (4)			*aacA4*, *strA*, *strB*, *Int1*, *3*′-*CS* (5)	
3	*Bacillus anthracis*	*Int1*, *3*′-*CS* (2)	*aacA4*, *aadA*, *strA*, *strB*, *Int1*, *3*′-*CS* (6)	*aacA4*, *aadA*, *strA*, *strB*, *Int1*, *3*′-*CS* (6)	*aacA4*, *aadA*, *strA*, *strB*, *Int1*, *3*′-*CS* (6)	*aacA4*, *aadA*, *strA*, *strB*, *Int1*, *3*′-*CS* (6)	*aacA4*, *aadA*, *strB* (3)
4	*Chryseobacterium lactis*	*aacA4*, *strA*, *strB*, *3*′-*CS* (4)	*aacA4*, *aadA*, *strA*, *strB*, *Int1*, *3*′-*CS* (6)	*aacA4*, *aadA*, *aadE*, *strA*, *strB*, *Int1*, *3*′-*CS* (7)	*aacA4*, *aadA*, *aphA1*, *strA*, *strB*, *Int1*, *3*′-*CS* (7)	*aacA4*, *aadA*, *strA*, *strB*, *Int1*, *3*′-*CS* (6)	
5	*Comamonas testosteroni*	*aacA4*, *strA*, *strB*, *3*′-*CS* (4)	*aacA4*, *strA*, *strB*, *Int1*, *3*′-*CS* (5)	*aacA4*, *strA*, *strB*, *Int1*, *3*′-*CS* (5)	*aacA4*, *strA*, *strB*, *Int1*, *3*′-*CS* (5)		
6	*Lactococcus chungangensis*	*strA*, *strB*, *3*′-*CS* (3)	*aacA4*, *aadA*, *strA*, *strB*, *Int1*, *3*′-*CS* (6)	*aacA4*, *aadA*, *aadB*, *aadE*, *strA*, *strB*, *Int1*, *3′*-*CS* (8)	*aacA4*, *aadA*, *aadE strA*, *strB*, *Int1*, *3*′-*CS* (7)	*aacA4*, *aadA*, *aadE strA*, *strB*, *Int1*, *3*′-*CS* (7)	*aacA4*, *aadA*, *aadE*, *strA*, *strB*, *Int1*, *3*′-*CS* (7)
7	*Lactococcus raffinolactis*	*strA*, *strB*, *3*′-*CS* (3)		*aacA4*, *aadA*, *aadE*, *strA*, *strB*, *Int1*, *3*′-*CS* (7)			
8	*Microbacterium maritypicum*				*aacA4*, *aadA*, *strA*, *strB*, *Int1*, *3*′-*CS* (6)	*aacA4*, *aadA*, *strA*, *strB*, *Int1*, *3*′-*CS* (6)	*aacA4*, *aadA*, *strA*, *strB*, *Int1*, *3*′-*CS* (6)
9	*Paracoccus yeei*	*Int1*, *3*′-*CS* (2)	*aacA4*, *aadA*, *aadB*, *strA*, *strB*, *Int1*, *3*′-*CS* (7)				
10	*Pseudoxanthomonas mexicana*	*aacA4*, *aadA*, *3*′-*CS* (3)			*aacA4*, *aadA*, *3*′-*CS* (3)	*aacA4*, *aadA*, *aadB*, *strB*, *Int1*, *3*′-*CS* (6)	*aacA4*, *aadA*, *aadB*, *strB*, *Int1*, *3*′-*CS* (6)
11	*Stenotrophomonas acidaminiphila*	*strB*, *Int1*, *3*′-*CS* (3)	*aacA4*, *aadA*, *aadB*, *aphA2*, *strA*, *strB*, *Int1*, *3*′-*CS* (8)				

Based on the MIC values, 40 distinguished streptomycin resistant isolates (includes 11 different strains) from various STM stages were selected for the ARGs analysis. Totally 11 ARGs were analyzed by the conventional PCR method

The number in parenthesis indicates that number of ARGs identified in STM resistant bacteria simultaneously and ‘_’ indicates the absence of ARGs in the corresponding bacteria

## Discussion

Although culture-independent approaches (meta-genomic) are widely used in recent times, bacterial antibiotic prevalence and resistant patterns are more accurate and evident only by the culture-dependent approaches (Czekalski et al. 2012). In the present culture-based long-term study, bacterial classes including *Gammaproteobacteria*, *Bacilli* and *Betaproteobacteria* were dominant in the reactor regardless of the presence or absence of STM (Fig. 2). In addition, major genera included *Aeromonas*, *Pseudomonas*, *Comamonas*, and *Bacillus* was dominant in all six STM exposures (Table 1; Additional file 1: Table S2). In our previous culture-independent study, bacterial classes including *Gammaproteobacteria*, *Betaproteobacteria* and *Bacteroidetes* dominated in the aerobic-biofilm reactors mainly receiving the wastewater containing STM, whereas the major genera included *Dokdonella*, *Pseudomonas*, *Desulfocapsa* and *Geobacter* (Deng et al. 2012). There are three possible reasons for this difference in bacterial compositions. The mixture of carbon sources used in the study, including glucose, tryptone, starch and sodium-carboxymethyl cellulose might have favored the different composition of genera. The culture-based approach might also produce favorable conditions for some bacterial genera in the WWTPs (Bramucci et al. 2003). On the other hand, anaerobic genera including *Desulfocapsa* and *Geobacter* might have come from the two anaerobic reactors used in the previous study, which received beta-lactam antibiotics at a minimal level (1.7–2.1 µg L^−1^). In this study, major bacterial genera and their populations were not disturbed even at the higher STM dose (25 and 50 mg L^−1^). It reveals that increasing STM doses did not affect the major bacterial diversity of the wastewater treatment system. Therefore, it is possible that they maintained their presence in the reactor by adopting antibiotic resistance in stepwise increasing doses of STM.

In the present study, bacterial isolates from the STM exposure reactor exhibited multi-resistance to other unrelated classes of antibiotics including macrolides, β-lactams, sulfonamides and tetracycline (Table 2; Additional file 1: Fig. S3). In our previous studies, similar multi-resistance observations were also described in wastewater systems accepting penicillin and oxytetracycline (Li et al. 2009, 2010). These related effects have also been observed in other sources such as clinical pathogens, chicken farms and aquatic systems (Tacao et al. 2014; Wong et al. 2014; Levy et al. 1976). According to previous other study on the overnight culture-based activated sludge samples exposed to minimal doses of STM (> 0.1 mg L^−1^) showed decreased bacterial multi-resistance by controlling the transfer of mobile genetic elements (Kim et al. 2014). In the current long-term study, minimum doses of STM (> 0.1 mg L^−1^) exhibited increased multi resistance (Fig. 3), whereas the higher doses of STM (> 25 mg L^−1^) exhibited stable multi-resistance. Similarly, on other hand, STM resistance also increased at minimum doses of STM (> 0.1 mg L^−1^) and was maintained at the higher doses of STM (> 25 mg L^−1^), whereas negligible STM resistance and multi-resistance were noted under no STM dose (0 mg L^−1^). We considered, therefore, that minimum doses of long-term exposure STM might induce significant MDR and STM resistance simultaneously, and the resistance could be able to persist even under higher doses of STM among the wastewater bacterial community.

It is interesting that the responses of every culturable wastewater bacterial strain to the stepwise increasing doses of STM were variable and unambiguous. *Chryseobacterium lactis* and *Comamonas testosteroni* naturally (0 mg L^−1^) possessed STM resistance genes (*strA* and *strB*) and a class I integron gene (*3*′-*CS*) (Table 3; Additional file 1: Table S7), and showed high MIC values against STM (> 256 µg mL^−1^) in the exposure without STM (Additional file 1: Table S5). Meanwhile, *Lactococcus chungangensis*, *Pseudoxanthomonas mexicana* and *Lactococcus raffinolactis* also naturally (0 mg L^−1^) possessed STM resistance genes (*strA* and *strB*) and an integron gene (*3*′-*CS*), but showed low MIC values against STM (< 6 µg mL^−1^) under the exposure without STM. Subsequently, however the above isolates obtained STM resistance under the lower doses of STM (0.1, 1, or 5 mg L^−1^). A similar previous observation suggested that the distribution of streptomycin MICs was influenced and controlled by STM resistance genes in *E. coli* (Sunde and Norstrom 2005). Furthermore, parameters such as the presence of selected gene cassettes and integron genes located on low copy number plasmids and their placement in 3′-conserved segments might lead to variable STM MIC results (Bryan 1984; Levesque 1994). We believe that the above parameters might be controlled by the threshold concentrations of selected antibiotics, which mostly fall in minimal dose ranges.

At the same time, *Aeromonas allosaccharophila* and *Aeromonas veronii* were not resistant to STM (< 8 µg mL^−1^) and did not contain any ARGs naturally (0 mg L^−1^). Subsequently, however, they became resistant to STM and ARGs were found (*aacA4*, *strA*, *strB*, *aadA*,* 3*′-*CS*, and *Int1*) in the corresponding STM exposures. This result indicates that STM resistance was promoted by stepwise increased STM concentrations, which might have occurred by the horizontal transfer of plasmids or transposons containing ARGs (Lekunberri et al. 2017; Van Overbeek et al. 2002; Rizzo et al. 2013; Czekalski et al. 2012). Interestingly, *Bacillus anthracis* was present and dominant in all six STM exposures (0–50 mg L^−1^), but the MIC results showed high sensitivity to STM (< 6 µg mL^−1^) and they were not grown in the growth agar media with low added STM dose (0.1 mg L^−1^). Surprisingly, this bacterial strain exhibited *strA*, *strB*, *aadA*, *aacA4*, *Int1* and* 3*′-*CS* as confirmed by conventional PCR. *Bacillus anthracis* is a spore-forming bacteria and the causative agent of anthrax disease. In general, spores of *Bacillus* sp. are dormant and resistant to unfavorable conditions such as extreme temperature, radiation, and antibiotics (Tetz et al. 2016; Severson et al. 2009; Nicholson et al. 2000). Previous findings revealed that bacterial spores can maintain their complete genome and antibiotic resistance genes might be involved in the horizontal transfer of ARGs to other bacterial species (Tetz and Tetz 2017; Barra-Carrasco et al. 2014). Furthermore, some ARGs are not expressed in the bacterial spores, but will be part of the resistome. In our study, we believed that spores might be produced under low STM exposure (0.1 mg L^−1^) and persist until the high STM exposure (> 25 mg L^−1^). However, further studies are needed to determine, whether the spores of *B. anthracis* play an important role in delivering STM resistance.

Non-pathogenic environmental bacteria such as *Lactococcus chungangensis*, *Lactococcus raffinolactis*, *Chryseobacterium lactis*, *Aeromonas allosaccharophila* and *Pseudoxanthomonas mexicana* showed the prevalence of *strA*, *strB*, *aacA4*, *IntI* and* 3*′-*CS* genes in the various STM exposures (0–50 mg L^−1^) and subsequently obtained STM resistance (> 256 µg mL^−1^) in the corresponding STM exposures. These results reveal that long-term increasing doses of STM can lead to non-pathogenic bacteria becoming perfect reservoirs of ARGs, which they might transfer to another bacterium via gene transfer, resulting in an abundance of MDR bacteria in WWTPs (Rizzo et al. 2013; Balcazar et al. 2015; Wellington et al. 2013).

In this study, most of the STM resistant strains from the reactor treated with various doses of STM (0–50 mg L^−1^) consisted of a 3′ conserved segment (*3*′-*CS*) of the clinically important class 1 integron gene and class I integrase (*Int1*) gene (85–92.5%). In addition, amino glycoside resistance genes *aacA4*, *strA*, *strB* and *aadA* (67.5–82.5%) were also distributed in most STM resistant strains from all STM exposures. These amino glycoside genes and integron genes were notably observed under the lowest STM dose (0.1 mg L^−1^). Therefore, minimum doses of STM might strongly induce the gene cassette of amino glycoside resistance genes among the wastewater bacterial community related to the clinical class I integron and class I integrase enzymes. Subsequently, these class I integrons might induce the dissemination of STM resistance to other wastewater bacteria through horizontal mobile gene transfer, which might then be maintained even under higher doses of STM (Mokracka et al. 2012; Masarikova et al. 2016; Said et al. 2016; Stalder et al. 2013; Nemec et al. 2004).

We also observed high prevalence of a non-STM resistance gene *aacA4* among the isolates harvested from the STM reactor exposure by involving stepwise increasing doses of STM (0–50 mg L^−1^). According to previous research, *aacA4* is an amino glycoside gene that encodes the enzyme, amino glycoside 6′-*N*-acetyl transferase (AAC(6′)-IbC) and confers resistance to antibiotics such as tobramycin, gentamycin and kanamycin (Nemec et al. 2004; Sacha et al. 2012). In the present study, however a prevalence of the *aacA4* gene was observed among 82.5% of STM isolates, equal to the prevalence of STM resistance genes *strA*, *strB*, *aadA* and *aadE* (15–82.5%). It is unclear, whether increased doses of STM modified the *aacA4* gene to encode STM-modifying enzymes or if it accelerated an unknown STM resistance mechanism. Further research is required to clarify this as well as antibiotic risk assessment in WWTPs. In conclusion, our present work reveals that the wide bacterial diversity among the indigenous bacteria from WWTPs exposed to long-term stepwise increasing doses of STM could become an enhanced and stable reservoir for the development of STM resistance. Here we concluded that stepwise increasing doses of antibiotics will expose a number of unanswered questions related to antibiotic resistance among the wastewater bacterial community. We therefore suggest that the current new approach could apply and extended in future studies to better understand the risk assessment of WWTPs.

## Additional file

**Additional file 1.** Tables and Figures.

## Abbreviations

STM: streptomycin
MDR: multi-drug resistance
MIC: minimum inhibitory concentration
WWTP: wastewater treatment plants
ARB: antibiotic resistant bacteria
ARGs: antibiotic resistance genes
PCR: polymerase chain reaction
HRT: hydraulic retention time
TSA: trypticase soya agar
CFUs: colony forming units
mM: milli molar
µM: micro molar
RDP: Ribosomal Database Project
NCBI: National Center for Biotechnology Information
CLSI: Clinical and Laboratory Standards Institute
AZ: azithromycin
TZ: ceftazidime
EF: enrofloxacin
SX: sulfamethoxazole
TC: tetracycline
TM: tobramycin
TGC: tigecycline
ETP: ertapenem
MH: Mueller Hinton
*3*′-*CS*: class I integron gene
*IntI*: class I integrase gene
Mg L^−1^: milligram per liter
