# Supplementary material for: Culture-based study on the development of antibiotic resistance in a biological wastewater system treating stepwise increasing doses of streptomycin
Source: AMB Express. 2018 Jan 25;8:12. doi: 10.1186/s13568-018-0539-x (PMC5783987; doi:10.1186/s13568-018-0539-x)
Supplement: Supplementary file 1 — Additional file 1. Tables and Figures. [file 13568_2018_539_MOESM1_ESM.docx]

**Additional file 1**

**Title: Culture-based study on the development of antibiotic resistance in a biological wastewater system treating stepwise increasing doses of Streptomycin**

**Ganesh-Kumar Selvaraj ^1^ · Zhe Tian ^1, 2^ · Hong Zhang ^1^ · Mohanapriya Jayaraman ^1^ · Min Yang ^1, 2^ · Yu Zhang ^1, 2 *^**

Ganesh-Kumar Selvaraj: ganesh_kumarbt@yahoo.co.in

Zhe Tian: tianzhescu@163.com

Hong Zhang: zhanghong20061014@163.com

Mohanapriya Jayaraman: biotech.jpriya@gmail.com

Min Yang: yangmin@rcees.ac.cn

Yu Zhang: zhangyu@rcees.ac.cn

***Correspondence:** Yu Zhang: zhangyu@rcees.ac.cn

^1^ State Key Laboratory of Environmental Aquatic Chemistry, Research Center for Eco-Environmental Sciences, Chinese Academy of Sciences, Beijing 100085, China

^2^ University of Chinese Academy of Sciences, Beijing 100049, China

**Contents**

Additional tables (Tables S1-S7)

Additional figure (S1-S4)

**
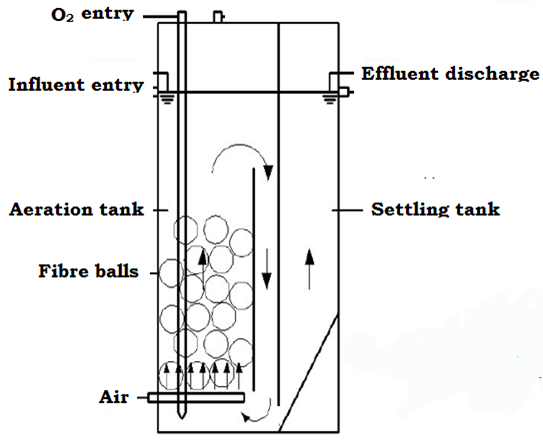
**

**Fig. S1:** A constructed aerobic long-term biofilm reactor with an effective volume of 2L and reactor filled with 18 fiber balls as bio-carriers.


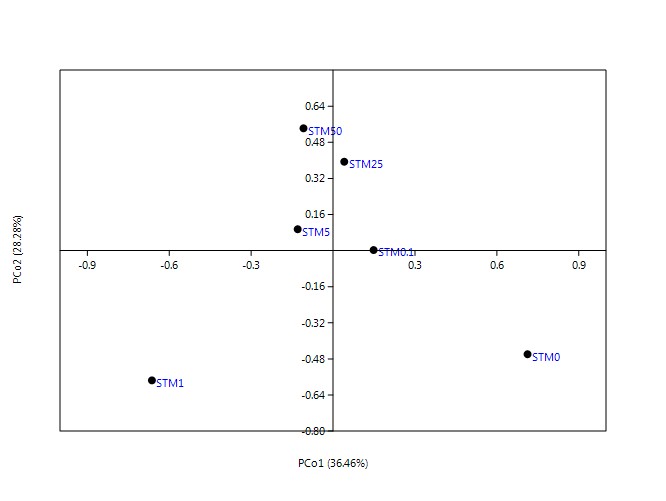


# Fig. S2: The variation in bacterial community composition between stepwise STM exposures. Principal coordinates analysis (PCoA) scatter plot shows the principal coordinate 1 (PCo1) versus principal coordinate 2 (PCo2). Percentages shown are percentages of variation explained by the components.

#

**Fig. S3:** Antibiotic resistance prevalence of bacterial isolates harvested from the various exposures of streptomycin. A total of 40 different bacterial strains (78 predominant bacterial isolates i.e. 13 isolates from each exposure) from various STM exposures were used for the MIC assay. The number of resistant strains among the total strains was considered for the percent calculations at every STM stages. (azithromycin (AZ), ceftazidime (TZ), enrofloxacin (EF), ertapenem (ETP), sulfamethoxazole (SX), tetracycline (TC), tigecycline (TGC),tobramycin (TM), and streptomycin (STM)).

**Fig. S4:** Number of types of amino glycoside resistant genes that simultaneously exhibited among the streptomycin resistant strains harvested from wastewater reactor that treated by gradually increased doses of streptomycin.

**Table S1.** Chemical composition of synthetic wastewater used in this study

| **Chemical ingredients** | **Concentration (mg·L^-1^)^1^** |
| --- | --- |
| Glucose (C_6_H_12_O_6_) | 150 |
| Soluble starch (C_6_H_10_O_5_)n | 100 |
| Sodium carboxymethyl cellulose (CMC) | 50 |
| Tryptone | 100 |
| Ammonium sulfate (NH_4_)_2_SO_4_ | 126 |
| Potassium dihydrogen phosphate (KH_2_PO_4_) | 31 |
| Sodium bicarbonate (NaHCO_3_) | 250 |
| ^2^Trace elements solution | 10 mL·L^-1^ |
| ^3^Streptomycin | 0, 0.1, 1, 5, 25 & 50 |

^1^All the corresponding chemicals were added with MilliQ water and prepared every time in freshly

^2^Trace elements solution：H_3_BO_3_, 50 mg·L^-1^; MnSO_4_·4H_2_O, 40 mg·L^-1^; ZnSO_4_·7H_2_O, 40 mg·L^-1^; Na_2_MoO_4_·4H_2_O, 20 mg·L^-1^; CuSO_4_·5H_2_O, 10 mg·L^-1^; CoCl_2_, 10 mg·L^-1^; KI, 10 mg·L^-1^

^3^Streptomycin solution was prepared freshly at every time and corresponding doses were mixed with methanol.

**Table S2**: Distribution of bacterial strains isolated from wastewater reactor treated by increased doses of streptomycin

| **No.** | **Bacterial Strains name** | **0mg L^-1^, STM** | **0.1mg L^-1^, STM** | **1mg L^-1^, STM** | **5mg L^-1^, STM** | **25mg L^-1^, STM** | **50mg L^-1^, STM** | **Total isolates** |
| --- | --- | --- | --- | --- | --- | --- | --- | --- |
| 1 | *Acidovorax temperans* | 1 |  |  |  |  |  | 1 |
| 2 | *Acinetobacter gyllenbergii* |  | 1 |  |  |  |  | 1 |
| 3 | *Acinetobacter johnsonii* |  | 1 |  |  |  |  | 1 |
| 4 | *Acinetobacter seohaensis* | 1 |  |  |  |  |  | 1 |
| 5 | *Acinetobacter sp.* |  |  | 2 | 2 |  | 1 | 5 |
| 6 | *Aeromonas caviae* |  |  |  | 1 |  |  | 1 |
| 7 | *Aeromonas allosaccharophila* | 1 |  |  |  | 2 | 2 | 5 |
| 8 | *Aeromonas media* | 2 |  |  |  |  |  | 2 |
| 9 | *Aeromonas salmonicida* |  |  |  | 1 |  |  | 1 |
| 10 | *Aeromonas veronii* | 1 | 2 |  |  | 2 |  | 5 |
| 11 | *Agromyces mediolanus* |  |  |  |  |  | 1 | 1 |
| 12 | *Arthrobacter nicotinovorans* |  |  |  |  |  | 2 | 2 |
| 13 | *Bacillus amyloliquefaciens* | 1 |  |  | 1 |  |  | 2 |
| 14 | *Bacillus anthracis* | 2 | 2 | 1 | 2 | 3 | 3 | 13 |
| 15 | *Bacillus aryabhattai* | 1 | 1 |  |  |  |  | 2 |
| 16 | *Bacillus cereus* |  | 1 | 1 |  |  |  | 2 |
| 17 | *Bacillus flexus* |  | 1 |  |  |  |  | 1 |
| 18 | *Bacillus safensis* | 1 | 1 |  |  |  |  | 2 |
| 19 | *Bacillus simplex* |  |  |  | 1 |  |  | 1 |
| 20 | *Bacillus stratosphericus* |  |  |  | 1 | 1 |  | 2 |
| 21 | *Bacillus thioparans* | 1 |  |  |  |  |  | 1 |
| 22 | *Bacillus toyonensis* | 1 |  |  |  |  |  | 1 |
| 23 | *Brevundimonas bullata* |  |  |  |  |  | 1 | 1 |
| 24 | *Brevundimonas terrae* | 1 |  |  |  |  |  | 1 |
| 25 | *Chitinimonas viridis* | 1 |  |  |  |  | 1 | 2 |
| 26 | *Chryseobacterium* sp |  | 1 | 0 |  |  |  | 2 |
| 27 | *Chryseobacterium lactis* | 1 | 2 | 1 | 2 | 2 |  | 8 |
| 28 | *Chryseobacterium rhizoplanae* | 1 |  |  |  |  |  | 1 |
| 29 | *Cloacibacterium normanense* | 2 |  |  |  |  |  | 2 |
| 30 | *Cloacibacterium rupense* | 1 |  |  |  |  |  | 1 |
| 31 | *Comamonas sp.* |  |  | 2 |  |  |  | 2 |
| 32 | *Comamonas testosteroni* | 2 | 1 | 3 | 1 |  |  | 7 |
| 33 | *Dechloromonas sp.* |  |  |  |  | 1 |  | 1 |
| 34 | *Delftia acidovorans* |  |  |  | 1 |  | 1 | 2 |
| 35 | *Delftia sp.* | 1 |  |  |  |  |  | 1 |
| 36 | *Delftia tsuruhatensis* |  |  | 2 |  |  |  | 2 |
| 37 | *Dyadobacter sp.* |  |  | 1 |  |  |  | 1 |
| 38 | *Flavobacterium hibernum* |  | 1 |  | 1 |  |  | 2 |
| 39 | *Flavobacterium sasangense* |  | 1 |  |  |  |  | 1 |
| 40 | *Hydrogenophaga pseudoflava* |  |  |  | 1 |  |  | 1 |
| 41 | *Hydrogenophaga taeniospiralis* |  |  |  |  | 1 |  | 1 |
| 42 | *Kocuria rhizophila* | 1 | 1 |  |  |  |  | 2 |
| 43 | *Lactococcus chungangensis* | 3 | 2 | 2 | 3 | 1 | 2 | 13 |
| 44 | *Lactococcus raffinolactis* | 1 |  | 1 |  |  |  | 2 |
| 45 | *Lelliottia amnigena* | 1 |  |  |  |  |  | 1 |
| 46 | *Leucobacter sp.* | 2 | 3 |  |  | 2 | 1 | 8 |
| 47 | *Lysinibacillus fusiformis* | 2 |  |  |  |  |  | 2 |
| 48 | *Lysobacter brunescens* | 1 |  |  |  |  |  | 1 |
| 49 | *Lysobacter sp.* | 1 |  |  |  |  |  | 1 |
| 50 | *Massilia timonae* |  |  |  |  |  | 1 | 1 |
| 52 | *Massilia varians* |  |  |  |  | 1 | 1 | 2 |
| 53 | *Microbacterium arabino.* | 1 |  | 1 |  |  |  | 1 |
| 54 | *Microbacterium lacticum* |  | 1 |  |  |  | 2 | 3 |
| 55 | *Microbacterium lacus* | 2 |  |  |  |  |  | 2 |
| 56 | *Microbacterium maritypicum* |  | 1 |  | 1 | 1 | 1 | 4 |
| 57 | *Micrococcus aloeverae* | 1 |  | 1 |  |  |  | 2 |
| 58 | *Micrococcus yunnanensis* | 1 |  |  |  |  |  | 1 |
| 59 | *Paracoccus yeei* | 1 | 1 |  |  |  |  | 2 |
| 60 | *Pseudomonas azotoformans* |  |  | 2 |  |  |  | 2 |
| 61 | *Pseudomonas japonica* | 1 |  |  |  |  |  | 1 |
| 62 | *Pseudomonas koreensis* |  |  | 1 |  |  |  | 1 |
| 63 | *Pseudomonas mendocina* |  |  |  |  | 1 |  | 1 |
| 64 | *Pseudomonas monteilii* |  |  |  | 1 |  |  | 1 |
| 65 | *Pseudomonas putida* |  |  | 1 |  |  |  | 1 |
| 66 | *Pseudoxanthomonas japonensis* |  |  |  | 1 |  | 1 | 2 |
| 67 | *Pseudoxanthomonas mexicana* | 2 |  |  | 1 | 2 | 1 | 6 |
| 68 | *Pseudoxanthomonas sp.* |  |  |  |  |  | 1 | 1 |
| 69 | *Raoultella ornithinolytica* |  | 1 |  |  |  |  | 1 |
| 70 | *Raoultella terrigena* |  |  | 1 | 1 | 1 |  | 3 |
| 71 | *Rhodococcus jialingiae* |  |  |  | 1 |  |  | 1 |
| 72 | *Rhodococcus yunnanensis* | 1 |  |  |  |  |  | 1 |
| 73 | *Rothia terrae* |  |  |  | 2 | 1 |  | 3 |
| 74 | *Runella zeae* | 1 |  |  |  |  |  | 1 |
| 75 | *Shewanella putrefaciens* |  |  | 1 |  |  |  | 1 |
| 76 | *Shinella zoogloeoides* | 1 |  |  |  |  |  | 1 |
| 77 | *Sphingobium xenophagum* | 1 |  |  |  |  |  | 1 |
| 78 | *Sphingopyxis chilensis* |  |  | 1 |  | 1 |  | 2 |
| 79 | *Sphingopyxis terrae* |  |  |  |  |  | 1 | 1 |
| 80 | *Staphylococcus capitis* | 1 |  |  |  |  |  | 1 |
| 81 | *Staphylococcus epidermidis* |  |  | 1 |  | 1 |  | 2 |
| 82 | *Staphylococcus warneri* | 2 |  |  |  |  |  | 2 |
| 83 | *Stenotrophomonas sp.* |  | 1 |  |  |  |  | 1 |
| 84 | *Stenotro. acidaminiphila* | 2 | 1 |  |  |  |  | 3 |
| 85 | *Streptococcus parauberis* | 1 |  |  |  |  |  | 1 |
| 86 | *Tahibacter aquaticus* |  |  |  |  | 1 |  | 1 |
| 87 | *Trichococcus flocculiformis* |  |  |  | 2 | 1 | 1 | 4 |
| 88 | *Trichococcus pasteurii* | 1 |  |  |  |  | 1 | 2 |
| 89 | *Wautersiella falsenii* | 1 |  |  |  |  |  | 1 |
| 90 | *Zoogloea caeni* |  |  |  | 1 |  |  | 1 |
|  | **Total bacterial isolates** | **56** | **28** | **26** | **29** | **26** | **26** | **191** |

**Table S3.** Bacterial community present in the control wastewater reactor

| **S. no** | **Name of bacterial strains** | **No of isolates** |
| --- | --- | --- |
| 1 | *Acidovorax temperans* | 1 |
| 2 | *Acinetobacter seohaensis* | 1 |
| 3 | *Aeromonas allosaccharophila* | 2 |
| 4 | *Aeromonas media* | 1 |
| 5 | *Aeromonas veronii* | 2 |
| 6 | *Bacillus amyloliquefaciens* | 1 |
| 7 | *Bacillus anthracis* | 2 |
| 8 | *Bacillus aryabhattai* | 1 |
| 9 | *Bacillus safensis* | 1 |
| 10 | *Bacillus simplex* | 1 |
| 11 | *Bacillus thioparans* | 1 |
| 12 | *Bacillus toyonensis* | 1 |
| 13 | *Brevundimonas bullata* | 1 |
| 14 | *Brevundimonas terrae* | 1 |
| 15 | *Chitinimonas viridis* | 1 |
| 16 | *Chryseobacterium* sp | 1 |
| 17 | *Chryseobacterium lactis* | 2 |
| 18 | *Chryseobacterium rhizoplanae* | 1 |
| 19 | *Cloacibacterium normanense* | 1 |
| 20 | *Cloacibacterium rupense* | 1 |
| 21 | *Comamonas testosteroni* | 2 |
| 22 | *Delftia sp.* | 1 |
| 23 | *Delftia tsuruhatensis* | 1 |
| 24 | *Hydrogenophaga taeniospiralis* | 1 |
| 25 | *Kocuria rhizophila* | 1 |
| 26 | *Lactococcus chungangensis* | 1 |
| 27 | *Lactococcus raffinolactis* | 1 |
| 28 | *Lelliottia amnigena* | 1 |
| 29 | *Leucobacter sp.* | 1 |
| 30 | *Lysinibacillus fusiformis* | 1 |
| 31 | *Lysobacter brunescens* | 1 |
| 32 | *Lysobacter sp.* | 1 |
| 33 | *Microbacterium arabino.* | 1 |
| 34 | *Microbacterium lacticum* | 1 |
| 35 | *Microbacterium lacus* | 1 |
| 36 | *Micrococcus aloeverae* | 1 |
| 37 | *Micrococcus yunnanensis* | 1 |
| 38 | *Paracoccus yeei* | 1 |
| 39 | *Pseudomonas japonica* | 1 |
| 40 | *Pseudoxanthomonas mexicana* | 2 |
| 41 | *Pseudoxanthomonas sp.* | 1 |
| 42 | *Rhodococcus yunnanensis* | 1 |
| 43 | *Runella zeae* | 1 |
| 44 | *Shinella zoogloeoides* | 1 |
| 45 | *Sphingobium xenophagum* | 1 |
| 46 | *Staphylococcus capitis* | 1 |
| 47 | *Staphylococcus epidermidis* | 1 |
| 48 | *Stenotrophomonas sp.* | 1 |
| 49 | *Stenotro. acidaminiphila* | 1 |
| 50 | *Streptococcus parauberis* | 1 |
| 51 | *Trichococcus flocculiformis* | 1 |
| 52 | *Wautersiella falsenii* | 1 |
|  | **Total isolates** | **58** |

***** Since the same inoculum and operating conditions of STM reactor was applied for the control reactor, an almost similar bacterial species were observed as present in 0 mg L^-1^ of STM reactor. So these stains from the control system were not used for the further analysis and not included in any of data results to avoid the result conflicts, but it was maintained as the same until the whole experimental period for monitoring other operating purposes and conditions. The colony forming units (CFU) of the control reactor was periodically monitored by the TSA plating and the same bacterial isolates that showing colony morphology was observed in the whole time, while no major different bacterial morphology was observed on the TSA media.

**Table S4:** The various classes of antibiotics used in MIC assay

| **Antibiotics** | **Class** | **Experimental range (µg/ml)** | **MIC method** |
| --- | --- | --- | --- |
| Azithromycin- AZ | Macrolides | 0.016 - 256 | Etest method |
| Ceftazidime- TZ | β-lactams | 0.016 - 256 | Etest method |
| Enrofloxacin- EF | Fluroquinolones | 0.002 - 32 | Etest method |
| Ertapenem- ETP | Carbapenem | 0.064 – 1024 | Etest method |
| Streptomycin -STM | Aminoglycosides | 1 - 1024 | 96-micro well plate method |
| Sulfamethoxazole- SX | Sulfonamides | 0.002 - 32 | Etest method |
| Tetracycline- TC | Tetracycline | 0.016 - 256 | Etest method |
| Tigecycline- TGC | Glycylcycline | 0.016 - 256 | Etest method |
| Tobramycin- TM | Aminoglycosides | 0.016 - 256 | Etest method |

**Table S5:** MIC values of bacterial isolates harvested from various exposures of streptomycin against the nine antibiotics.

| **S. No** | **Bacterial Strains name** | **STM**  **stages**  **(mg L^-1^)** | **AZ^1^**  **(.016-256 µg/ml)** | **TZ^1^**  **(.016-256 µg/ml)** | | **EF^1^**  **(.002-32 µg/ml)** | **ETP^1^**  **(.002-32 µg/ml)** | **SX^1^**  **(.064-1024 µg/ml)** | | **TC^1^**  **(.016-256 µg/ml)** | | **TGC^1^**  **(.016-256 µg/ml)** | **TM^1^**  **(.016-256 µg/ml)** | **STM^2^**  **(1-1024**  **µg/ml)** |
| --- | --- | --- | --- | --- | --- | --- | --- | --- | --- | --- | --- | --- | --- | --- |
| 1 | *Acinetobacter sp.* | 1mg | 162.0 | | 1.0 | 0.75 | 0.50 | | 0.38 | | 24.0 | 0.50 | 0.125 | >1024 |
|  |  | 5mg | 162.0 | | 2.0 | 1.0 | 0.75 | | 0.38 | | 16.0 | 0.25 | 0.18 | 1024 |
|  |  | 50mg | 128.0 | | 3.0 | 2.0 | 1.0 | | 0.38 | | 16.0 | 0.25 | 0.125 | 512 |
| 2 | *Aeromonas allosaccharophila* | 0mg | 1.0 | 0.064 | | 1.5 | 2.0 | - | | 12.0 | | 1.5 | 2.0 | 6 |
|  |  | 25mg | 3.0 | 7.5 | | 1.0 | 0.25 | - | | 64.0 | | 1.0 | 16.0 | 1024 |
|  |  | 50mg | 12.0 | 64.0 | | 1.0 | 0.047 | - | | 16.0 | | 0.125 | 16.0 | >1024 |
| 3 | *Aeromonas veronii* | 0mg | 0.38 | 0.064 | | 0.125 | 0.19 | - | | 8.0 | | 0.19 | 1.0 | 6 |
|  |  | 0.1mg | 48.0 | 4.5 | | 1.0 | 0.25 | - | | 1.5 | | 0.19 | 6.0 | 256 |
|  |  | 25mg | 16.0 | 48.0 | | 0.50 | 0.023 | - | | 0.75 | | 0.38 | 6.0 | >1024 |
| 4 | *Agromyces mediolanus* | 50mg | 1 | | 1.5 | 4 | 1 | | 0.50 | | 16 | 0.25 | 128 | 256 |
| 5 | *Bacillus amyloliquefaciens* | 0mg | 0.19 | - | | 0.047 | 0.19 | 3.0 | | 3.0 | | 0.094 | 0.047 | 4 |
|  |  | 5mg | 0.19 | - | | 0.094 | 0.19 | 6.0 | | 4.0 | | 0.094 | 0.032 | 4 |
| 6 | *Bacillus anthracis* | 0mg | 0.50 | - | | 0.38 | 0.50 | 1.5 | | 1.5 | | 0.50 | 1.0 | 2 |
|  |  | 0.1mg | 0.50 | - | | 0.50 | 0.50 | 3.0 | | 2.0 | | 0.125 | 1.5 | 2 |
|  |  | 1mg | 0.50 | - | | 0.50 | 0.50 | 16.0 | | 2.0 | | 0.125 | 1.5 | 6 |
|  |  | 5mg | 0.125 | - | | 0.50 | 0.064 | 6.0 | | 4.0 | | 0.125 | 1.5 | 6 |
|  |  | 25mg | 0.50 | - | | 0.25 | 0.064 | 6.0 | | 4.0 | | 0.094 | 1.5 | 6 |
|  |  | 50mg | 0.50 | - | | 0.125 | 0.023 | 1.5 | | 2.0 | | 0.094 | 0.023 | 1 |
| 7 | *Bacillus cereus* | 0.1mg | 1.5 | | - | 0.52 | 0.50 | | 1.0 | | 1.5 | 0.094 | 3.0 | 4 |
|  |  | 1mg | 0.19 | | - | 0.023 | 0.023 | | 1.5 | | 2.0 | 0.064 | 0.094 | 4 |
| 8 | *Bacillus stratosphericus* | 5mg | 1.5 | | - | 0.064 | 0.094 | | 0.25 | | 0.75 | 0.064 | 0.094 | 1 |
|  |  | 25mg | 1 | | - | 0.125 | 0.032 | | 1.5 | | 2 | 0.064 | 0.064 | 1 |
| 9 | *Brevundimonas bullata* | 50mg | 2.0 | | - | 0.19 | 0.032 | | - | | 0.25 | 0.094 | 32.0 | 4 |
| 10 | *Chryseobacterium lactis* | 0mg | 3.0 | 3.0 | | 0.094 | - | 24.0 | | 8.0 | | 1.0 | - | 256 |
|  |  | 0.1mg | 48.0 | 32.0 | | 0.094 | - | 384.0 | | 16.0 | | 1.5 | - | 512 |
|  |  | 1mg | 48.0 | 48.0 | | 0.19 | - | 512.0 | | 16.0 | | 0.047 | - | >1024 |
|  |  | 5mg | 18.0 | 3.0 | | 0.125 | - | 512.0 | | 32.0 | | 1.5 | - | 256 |
|  |  | 25mg | 1.5 | 0.125 | | 0.50 | - | 192.0 | | 16.0 | | 0.38 | - | 256 |
| 11 | *Comamonas sp.* | 1mg | 1.0 | | 0.38 | 0.032 | 0.125 | | 3.0 | | 0.75 | 0.047 | 4.0 | 512 |
| 12 | *Comamonas testosteroni* | 0mg | 3.0 | 0.75 | | 0.094 | 1.0 | - | | 0.25 | | 0.19 | 1.5 | 256 |
|  |  | 0.1mg | 64.0 | 12.0 | | 0.19 | 0.125 | - | | 16.0 | | 0.047 | 8.0 | 256 |
|  |  | 1mg | 64.0 | 48.0 | | 0.125 | 0.38 | - | | 32.0 | | 0.19 | 48.0 | >1024 |
|  |  | 5mg | 0.25 | 64.0 | | 0.094 | 0.50 | - | | 32.0 | | 0.25 | 16.0 | >1024 |
| 13 | *Flavobacterium hibernum* | 0.1mg | 1.0 | | 0.125 | 0.38 | 0.25 | | - | | 0.25 | 0.094 | 0.125 | 1 |
|  |  | 5 mg | 1.0 | | 0.25 | 0.38 | 0.25 | | - | | 0.75 | 0.047 | 0.18 | 12 |
| 14 | *Kocuria rhizophila* | 0mg | 3.0 | 0.032 | | 1.0 | 0.023 | 0.19 | | 0.19 | | 0.047 | 3.0 | 6 |
|  |  | 0.1mg | 0.064 | 0.50 | | 3.0 | 0.064 | 0.50 | | 0.25 | | 0.064 | 3.0 | 32 |
| 15 | *Lactococcus chungangensis* | 0mg | 0.094 | 6.0 | | 0.38 | 0.047 | 3.0 | | 0.25 | | 0.047 | 0.75 | 1 |
|  |  | 0.1mg | 0.125 | 32.0 | | 0.19 | 0.094 | 3.0 | | 0.25 | | 0.047 | 0.25 | 4 |
|  |  | 1mg | 0.064 | 32.0 | | 0.38 | 0.032 | 24.0 | | 0.25 | | 0.047 | 0.50 | 4 |
|  |  | 5mg | 0.75 | 96.0 | | 0.38 | 0.032 | 64.0 | | 0.25 | | 0.047 | 0.064 | 256 |
|  |  | 25mg | 0.19 | 16.0 | | 0.19 | 0.016 | 6.0 | | 0.75 | | 0.047 | 1.5 | 128 |
|  |  | 50mg | 0.19 | 16.0 | | 0.038 | 0.125 | 4.0 | | 0.25 | | 0.047 | 1.0 | 128 |
| 16 | *Lactococcus raffinolactis* | 0mg | 0.125 | 16.0 | | 0.25 | 0.047 | 4.0 | | 0.38 | | 0.047 | 0.75 | 4 |
|  |  | 1mg | 0.125 | 48.0 | | 0.50 | 0.047 | 6.0 | | 0.38 | | 0.064 | 0.50 | 256 |
| 17 | *Leucobacter sp.* | 0mg | 4.0 | 12.0 | | 0.38 | 0.25 | - | | 2.0 | | 0.094 | 6.0 | 4 |
|  |  | 0.1mg | 4.0 | 6.0 | | 0.047 | 0.047 | - | | 16.0 | | 0.094 | 3.0 | 4 |
|  |  | 25mg | 24.0 | 0.38 | | 0.38 | 0.047 | - | | 64.0 | | 0.094 | 1.5 | 2 |
|  |  | 50mg | 4.0 | 0.19 | | 0.38 | 0.012 | - | | 8.0 | | 0.094 | 1.5 | 2 |
| 18 | *Microbacterium lacticum* | 0.1mg | 24.0 | | - | 0.38 | 0.25 | | - | | 3.0 | 0.064 | 24.0 | >512 |
|  |  | 50mg | 64.0 | | - | 0.50 | 0.25 | | - | | 4.0 | 0.064 | 16.0 | >1024 |
| 19 | *Microbacterium maritypicum* | 0.1mg | 24.0 | | - | 1.0 | 16.0 | | 3.0 | | 3.0 | 0.094 | 8.0 | >1024 |
|  |  | 5mg | >256.0 | | - | 1.0 | 16.0 | | 16.0 | | 16.0 | 0.094 | 8.0 | >512 |
|  |  | 25mg | >256.0 | | - | 0.50 | >32 | | 128.0 | | 24.0 | 0.047 | 8.0 | 64 |
|  |  | 50mg | 48.0 | | - | 0.50 | 16.0 | | 1.2 | | 1.2 | 0.047 | 8.0 | 1024 |
| 20 | *Paracoccus yeei* | 0mg | 0.094 | 6.0 | | 0.125 | 0.064 | 8.0 | | 0.19 | | 0.047 | 1.0 | 6 |
|  |  | 0.1mg | 0.047 | 6.0 | | 0.064 | 0.047 | 48.0 | | 0.125 | | 0.047 | 0.75 | 512 |
| 21 | *Pseudomonas azotoformans* | 1mg | 8.0 | | 1.0 | 8.0 | 1.0 | | 6.0 | | 24.0 | 1.5 | - | - |
| 22 | *Pseudomonas koreensis* | 1mg | 32.0 | | 16.0 | 0.75 | 1.0 | | 6.0 | | 3.0 | 1.0 | 0.19 | 12 |
| 23 | *Pseudomonas putida* | 1mg | 24.0 | | 1.5 | 1.5 | 1.0 | | - | | 4.0 | 4.0 | 4.0 | 512 |
| 24 | *Pseudoxanthomonas sp.* | 50mg | 0.50 | | 0.25 | 0.75 | 0.008 | | 0.125 | | 0.50 | 0.023 | 1.0 | 4 |
| 25 | *Pseudoxanthomonas mexicana* | 0mg | 0.50 | 0.125 | | 0.016 | 0.50 | - | | 1.0 | | 0.19 | 12.0 | 6 |
|  |  | 5mg | 0.38 | 0.19 | | 0.023 | 0.038 | - | | 0.75 | | 0.19 | 64.0 | 256 |
|  |  | 25mg | 0.25 | 0.38 | | 0.047 | 0.75 | - | | 1.0 | | 0.125 | 128.0 | >1024 |
|  |  | 50mg | 0.25 | 0.19 | | 0.016 | 0.50 | - | | 1.0 | | 0.125 | 128.0 | >1024 |
| 26 | *Raoultella terrigena* | 1mg | 6.0 | | 0.125 | 0.75 | 0.006 | | 16.0 | | 3.0 | 0.08 | 0.75 | 512 |
|  |  | 5mg | 4.0 | | 0.25 | 0.047 | 0.50 | | 16.0 | | 1.5 | 0.50 | 0.50 | >1024 |
|  |  | 25mg | 8.0 | | 0.25 | 0.064 | 0.50 | | 48.0 | | 1.5 | 0.50 | 0.50 | >1024 |
| 27 | *Rothia terrae* | 5mg | 16.0 | | 96.0 | 0.38 | 1.0 | | 0.125 | | 0.75 | 0.50 | 2.0 | 64 |
|  |  | 25mg | 1.0 | | 12.0 | 0.25 | 1.0 | | 0.125 | | 0.50 | 0.50 | 2.0 | 1 |
| 28 | *Stenotrophomonas acidaminiphila* | 0mg | 0.50 | 0.50 | | 0.016 | 0.094 | - | | 0.25 | | 0.25 | 0.25 | 4 |
|  |  | 0.1mg | 6.0 | 0.50 | | 0.032 | 1.0 | - | | 12.0 | | 0.25 | 0.50 | 512 |
| 29 | *Sphingopyxis chilensis* | 1mg | - | | 1.0 | - | 0.006 | | - | | 0.75 | 0.023 | 2.0 | >1024 |
|  |  | 25mg | - | | 1.5 | - | 0.50 | | - | | 0.75 | 0.50 | 4.0 | 1 |
| 30 | *Sphingopyxis terrae* | 50mg | - | | 1.0 | - | 0.50 | | - | | 1.5 | 0.50 | 0.50 | 32 |
| 31 | *Trichococcus flocculiformis* | 5mg | - | | 0.38 | 0.19 | 0.008 | | 3.0 | | 24.0 | 0.023 | 1.0 | 4 |
|  |  | 25mg | - | | 0.38 | 0.19 | 0.50 | | 2.0 | | 16.0 | 0.023 | 1.5 | 4 |
|  |  | 50mg | - | | 0.38 | 0.125 | 0.50 | | 4.0 | | 4.0 | 0.38 | 1.0 | 8 |

^1^ Antimicrobial susceptibility testing was estimated by agar diffusion method (Etest gradient epsilometer technique; duplicates) and incubated at 30ºC for 24h.

^2^ Antimicrobial susceptibility testing was estimated two-fold serial dilution method (since no commercial Estrip; triplicates) and incubated at 30ºC for 24h.

“-“ represents ‘no response’ of bacteria against the antibiotics and it was considered as ‘sensitive’ for our MIC interpretation

A total of 31 bacterial strains (78 isolates) that dominated in the various STM stages were used for the MIC assay. Abbreviations: azithromycin (AZ), ceftazidime (TZ), enrofloxacin (EF), ertapenem (ETP), sulfamethoxazole (SX), tetracycline (TC), tigecycline (TGC), tobramycin (TM), and streptomycin (STM)).

* Since no present CLSI guidelines for environmental non-pathogenic bacteria, the MIC pattern was grouped as sensitive (0.016–12μg/mL), resistant (13–64μg/mL), and high resistant (64–256μg/mL) (Lundstrom *et al.,* 2016; Popowska *et al.,* 2012). However, reported cut-off value of streptomycin (>8μg/mL) was considered for the STM resistance (Sunde and Norstrom, 2005).

**Table S6.** The primers for amino glycoside genes, integron genes and 16SrRNA gene used in the study

| **Primer** | **Forward primer sequence** | **Reverse primer sequence** | **bp** | **Annealing temp(℃)** | **Resistant to*** | **References** |
| --- | --- | --- | --- | --- | --- | --- |
| *aacA4* | TTGCGATGCTCTATGAGTGGCTA | CTCGAATGCCTGGCGTGTTT | 482 | 55.0 | Tm, Gen, Kan | 4 |
| *aadA* | AAATTCTTCCAACTGATCTGCG | CCTGAACAGGATCTATTTGAGGC | 276 | 58.3 | STM | 10 |
| *aadB* | TGGTGGTACTTCATCGGCATA | GTTACTTGACTGCGAACCTGCT | 175 | 54.1 | Tm, Kan | 10 |
| *aadE* | GATCTTACCTTATTGCCCTTGGA | GCGCTTGGCTTTCTTACATG | 143 | 53.1 | STM, Sp | 10 |
| *aphA1* | AAACGTCTTGCTCGAGGC | CAAACCGTTATTCATTCGTGA | 500 | 50.7 | Tm, Kan | 7 |
| *aphA2* | GATTGAACAAGATGGATTGC | CCATGATGGATACTTTCTCG | 347 | 50 | Kan, Neo | 11 |
| *strA* | CCTGGTGATAACGGCAATTC | CCAATCGCAGATAGAAGGC | 546 | 52.7 | STM | 2 |
| *strB* | ATCGTCAAGGGATTGAAACC | GGATCGTAGAACATATTGGC | 509 | 50.8 | STM | 2 |
| *aac(3)-II* | TGAAACGCTGACGGAGCCTC | GTCGAACAGGTAGCACTGAG | 370 | 55.8 | Gen, Tm, Kan | 6 |
| *Intl1* | CCTCCCGCACGATGATC | TCCACGCATCGTCAGGC | 280 | 56.0 | encodes Class I integrase | 1 |
| *3-CS* | CAAACTATCAGGTCAAGTCTGCT | GTCCGAACTCCACGACGTCTGATC | 550 | 56.0 | encodes Class I integron | 8 |
| *16SrRNA* | AGA GTT TGA TCC TGG CTC AG | TAC GGY TAC CTT GTT ACG ACT T | 1500 | 52.0 | encodes 16SrRNA | 12 |

*Tm-Tobramycin, Gen-Gentamycin, kan-Kanamycin, STM-streptomycin, Sp-spectinomycin, Neo-neomycin,

**Table S7.** Presence and distributions of antibiotic resistant genes (ARGs) and integron genes in various stages of streptomycin resistant bacteria

| **S. No** | **Bacterial Strains*** | **STM**  **(mg/L)** | ***aac (3)-II*** | ***aacA4*** | ***aadA*** | ***aadB*** | ***aadE*** | ***aphA1*** | ***aphA2*** | ***strA*** | ***strB*** | ***Int1*** | ***3’-CS*** |
| --- | --- | --- | --- | --- | --- | --- | --- | --- | --- | --- | --- | --- | --- |
| 1 | *Aeromonas allosaccharophila* | 0mg | - | - | - | - | - | - | - | - | - | - | - |
|  |  | 25mg | - | +++ | - | - | - | - | - | + | + | +++ | +++ |
|  |  | 50mg | - | +++ | + | - | - | - | - | + | + | +++ | +++ |
| 2 | *Aeromonas veronii* | 0mg | - | - | - | - | - | - | - | - | - | - | - |
|  |  | 0.1mg | - | + | +++ | - | - | - | - | - | - | +++ | +++ |
|  |  | 25mg | - | +++ | - | - | - | - | - | + | + | +++ | +++ |
| 3 | *Bacillus anthracis* | 0mg | - | - | - | - | - | - | - | - | - | ++ | + |
|  |  | 0.1mg | - | + | + | - | - | - | - | + | + | ++ | + |
|  |  | 1mg | - | ++ | + | - | - | - | - | + | + | ++ | + |
|  |  | 5mg | - | ++ | + | - | - | - | - | + | + | ++ | + |
|  |  | 25mg | - | +++ | + | - | - | - | - | + | + | +++ | ++ |
|  |  | 50mg | - | ++ | + | - | - | - | - | - | + | - | - |
| 4 | *Chryseobacterium lactis* | 0mg | - | ++ | - | - | - | - | - | + | + | - | + |
|  |  | 0.1mg | - | ++ | + | - | - | + | - | ++ | ++ | + | + |
|  |  | 1mg | - | +++ | +++ | - | +++ | - | - | +++ | +++ | ++ | ++ |
|  |  | 5mg | - | + | + | - | - | - | - | ++ | ++ | + | + |
|  |  | 25mg | - | + | + | - | - | - | - | + | + | + | + |
| 5 | *Comamonas testosteroni* | 0mg | - | +++ | - | - | - | - | - | +++ | +++ | - | +++ |
|  |  | 0.1mg | - | +++ | - | - | - | + | - | +++ | +++ | +++ | +++ |
|  |  | 1mg | - | +++ | - | - | - | - | - | +++ | +++ | +++ | ++ |
|  |  | 5mg | - | +++ | - | - | - | - | - | +++ | +++ | +++ | ++ |
| 6 | *Lactococcus chungangensis* | 0mg | - | - | - | - | - | - | - | + | + | - | + |
|  |  | 0.1mg | - | + | + | - | - | - | - | + | + | + | + |
|  |  | 1mg | - | +++ | +++ | + | +++ | - | - | ++ | ++ | + | ++ |
|  |  | 5mg | - | + | + | - | + | - | - | + | + | + | + |
|  |  | 25mg | - | + | + | - | +++ | - | - | + | + | + | + |
|  |  | 50mg | - | + | ++ | - | +++ | - | - | ++ | ++ | + | + |
| 7 | *Lactococcus raffinolactis* | 0mg | - | - | - | - | - | - | - | + | + | - | + |
|  |  | 1mg | - | + | + | - | +++ | - | - | ++ | ++ | + | + |
| 8 | *Paracoccus yeei* | 0mg | - | - | - | - | - | - | - | - | - | + | + |
|  |  | 0.1mg | - | ++ | + | + | - | - | - | + | ++ | + | + |
| 9 | *Pseudoxanthomonas mexicana* | 0mg | - | + | + | - | - | - | - | - | - | - | + |
|  |  | 5mg | - | + | + | - | - | - | - | - | - | - | ++ |
|  |  | 25mg | - | +++ | +++ | +++ | - | - | - | +++ | ++ | ++ | +++ |
|  |  | 50mg | - | +++ | +++ | +++ | - | - | - | +++ | +++ | ++ | +++ |
| 10 | *Stenotrophomonas acidaminiphila* | 0mg | - | - | +++ | +++ | - | - | +++ | - | +++ | ++ | +++ |
|  |  | 0.1mg | - | + | +++ | +++ | - | - | +++ | + | + | ++ | +++ |
| 11 | *Microbacterium maritypicum* | 5mg | - | ++ | + | - | - | - | - | + | + | + | + |
|  |  | 25mg | - | + | +++ | - | - | - | - | ++ | + | + | ++ |
|  |  | 50mg | - | ++ | + | - | - | - | - | ++ | ++ | + | + |

*11 significant STM resistant bacterial strains (40 isolates) that identified from MIC assay were subjected to resistant gene prevalence analysis.

“-“ means no DNA band in agarose gel (1.5%)

“+” (very low), “++” (medium) and “+++” (high) represent the intensity of specific band in agarose gel

**REFERENCES**

1. Goldstein C, Lee MD, Sanchez S, Hudson C, Phillips B, Register B, Grady M, Liebert C, Summers AO, White DG, Maurer JJ. Incidence of class 1 and 2 integrases in clinical and commensal bacteria from livestock, companion animals, and exotics. Antimicrob Agents Chemother, 2001, 45(3): 723-726.
2. Lanz R, Kuhnert P, Boerlin P. Antimicrobial resistance and resistance gene determinants in clinical *Escherichia coli* from different animal species in Switzerland. Vet Microbiol, 2003, 91(1): 73-84.
3. Lundstrom, S.V., Östman, M., Bengtsson-Palme, J., Rutgersson, C., Thoudal, M., Sircar, T., Blanck, P., Eriksson, K.M., Tysklind, M., Flach, C., and Larsson, D.G.J., 2016. Minimal selective concentrations of tetracycline in complex aquatic bacterial biofilms. Science of the Total Environment. 553, 587–595
4. Park CH, Robicsek A, Jacoby GA, Sahm D, Hooper DC. Prevalence in the United States of *aac(6')-Ib-cr* encoding a ciprofloxacin-modifying enzyme. Antimicrob Agents Chemother, 2006, 50(11): 3953-3955.
5. Popowska P., Rzeczycka M., Miernik A, Krawczyk-Balska A., Walsh F., and Duffy B., 2012. Influence of Soil Use on Prevalence of Tetracycline, Streptomycin, and Erythromycin Resistance and Associated Resistance Genes Antimicrobial Agents and Chemotherapy. 56 (3) 1434–1443.
6. Sandvang D, Aarestrup FM. Characterization of aminoglycoside resistance genes and class 1 integrons in porcine and bovine gentamicin-resistant *Escherichia* *coli*. Microb Drug Resist, 2000, 6(1): 19-27.
7. Shahada F, Chuma T, Tobata T, Okamoto K, Sueyoshi M, Takase K. Molecular epidemiology of antimicrobial resistance among *Salmonella enterica* serovar Infantis from poultry in Kagoshima, Japan. Int J Antimicrob Agents, 2006, 28(4): 302-307.
8. Stalder, T., Barraud, O., Jove, T., Casellas, M., Gaschet, M., Dagot, C., and CecilePloy, M., 2013. Quantitative and qualitative impact of hospital effluent on dissemination of the integron pool, The ISTME Journal 8(4):768-77.
9. Sunde, M., and Norstrom, M., 2005. The genetic background for streptomycin resistance in Escherichia coli influences the distribution of MICs. Journal of Antimicrobial Chemotherapy. 56, 87–90.
10. Tian, Z, Zhang, Y, Yu, B, Yang, M., Changes of resistome, mobilome and potential hosts of antibiotic resistance genes during the transformation of anaerobic digestion from mesophilic to thermophilic, 2016, 98, 261-269
11. Travis, R. M., Gyles, C. L., Reid-STMith, R., Poppe, C., McEwen, S. A., Friendship, R., Janecko, N., and Boerlin, P., 2006. Chloramphenicol and kanamycin resistance among porcine *Escherichia coli* in Ontario. Journal. of Antimicrobial Chemotherapy. 58 (1), 173-177.
12. Weisburg, W.G., Barns, S.M., Pelletier, D.A., and Lane, D.J., 1991. 16S ribosomal DNA amplification for phylogenetic study, Journal of Bacteriology. 173(2) 697-703.
